# Supplementary material for: Autoxidation Enhances Anti-Amyloid Potential of Flavone Derivatives
Source: Antioxidants (Basel). 2021 Sep 7;10(9):1428. doi: 10.3390/antiox10091428 (PMC8465893; doi:10.3390/antiox10091428)
Supplement: Supplementary file 1 [file antioxidants-10-01428-s001.zip › antioxidants-1354908-supplementary.pdf]

Supplementary Materials

Autoxidation Enhances Anti-Amyloid Potential of Flavone Derivatives

Andrius Sakalauskas, Mantas Ziaunys, Ruta Snieckute and Vytautas Smirnovas *

Life Sciences Center, Institute of Biotechnology, Vilnius University, LT-10257 Vilnius, Lithuania; andrius.sakalauskas@gmc.vu.lt (A.S.); mantas.ziaunys@gmail.com (M.Z.); r.snieckute@gmail.com (R.S.)
* Correspondence: vytautas.smirnovas@bti.vu.lt

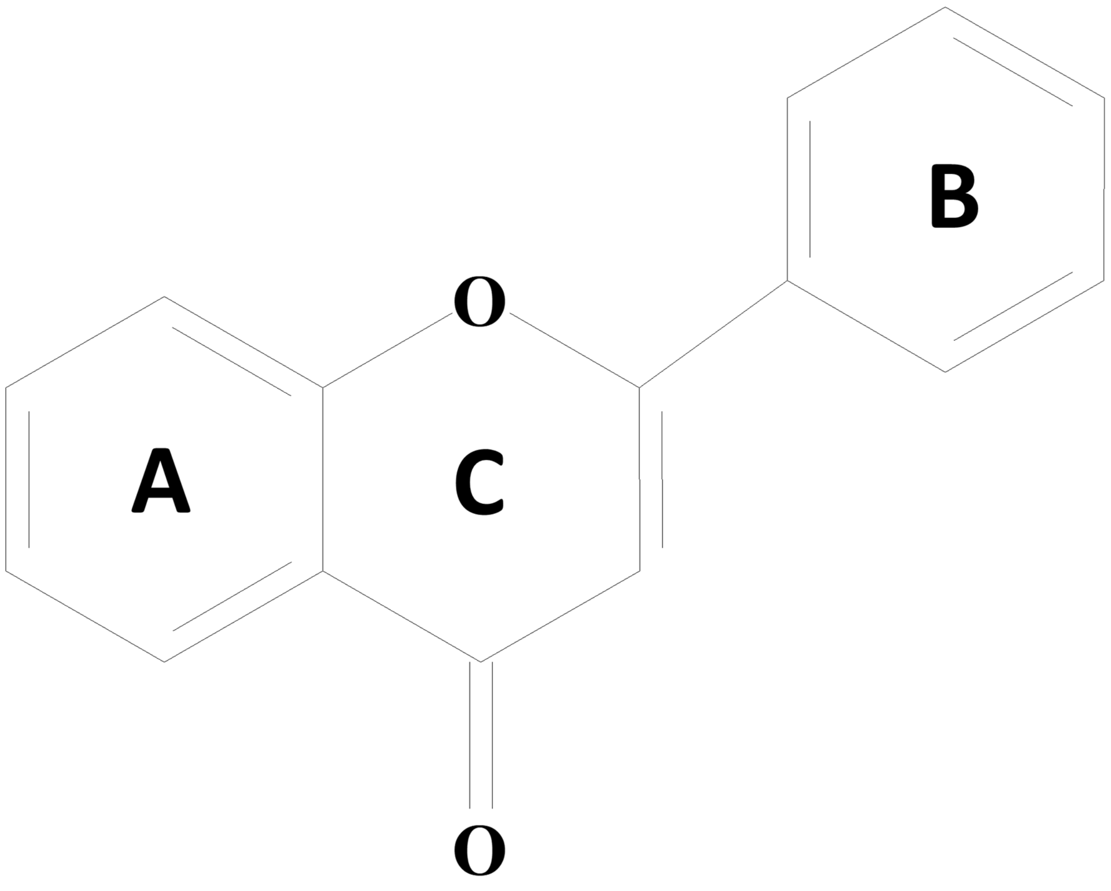

Figure S1. Flavone structure. Structure was drawn using Chromdraw program.

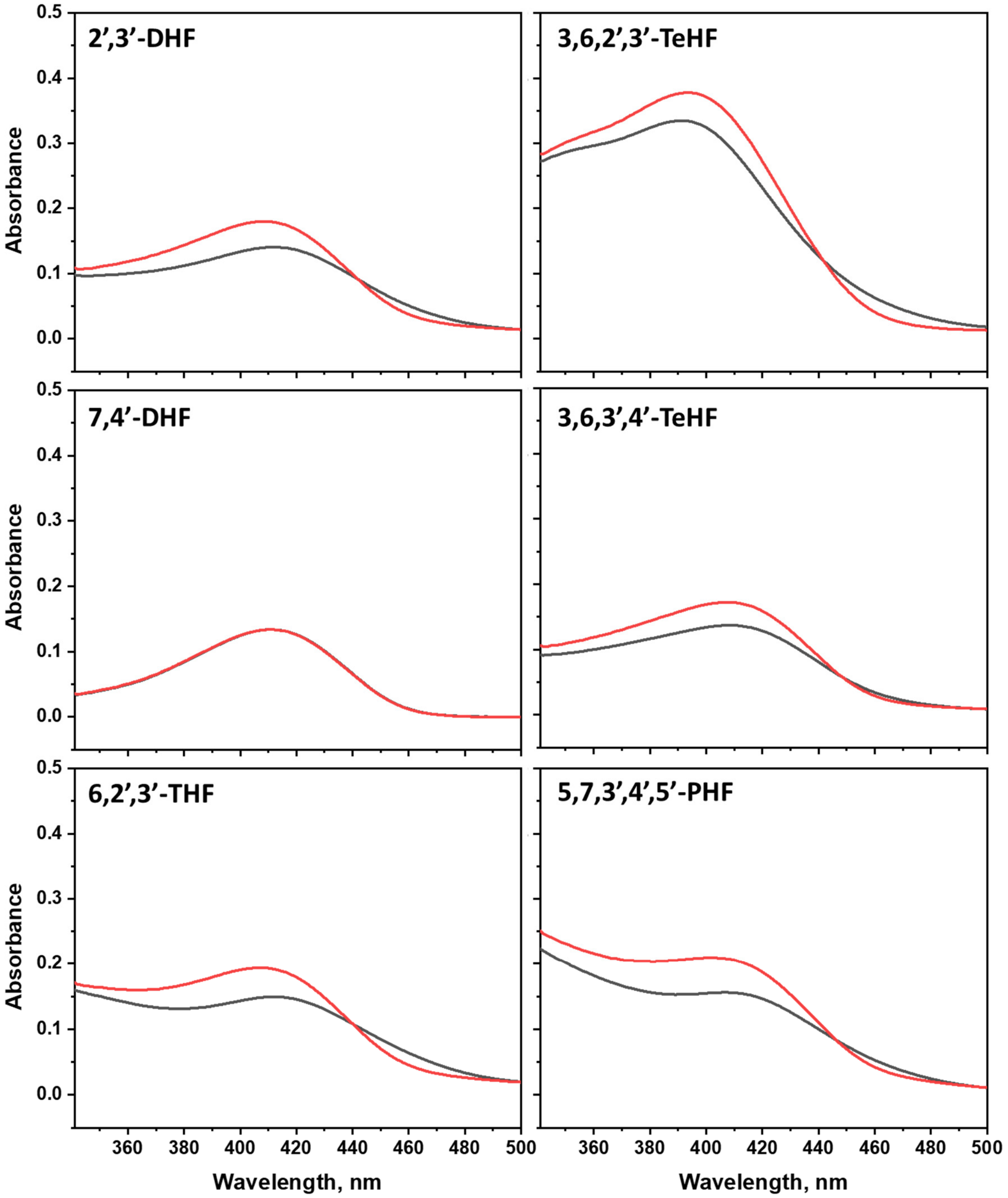

Figure S2. Absorbance spectra of incubated flavone mixed with ThT (red) and combined spectra of incubated flavone and ThT when they are scanned separately (black).

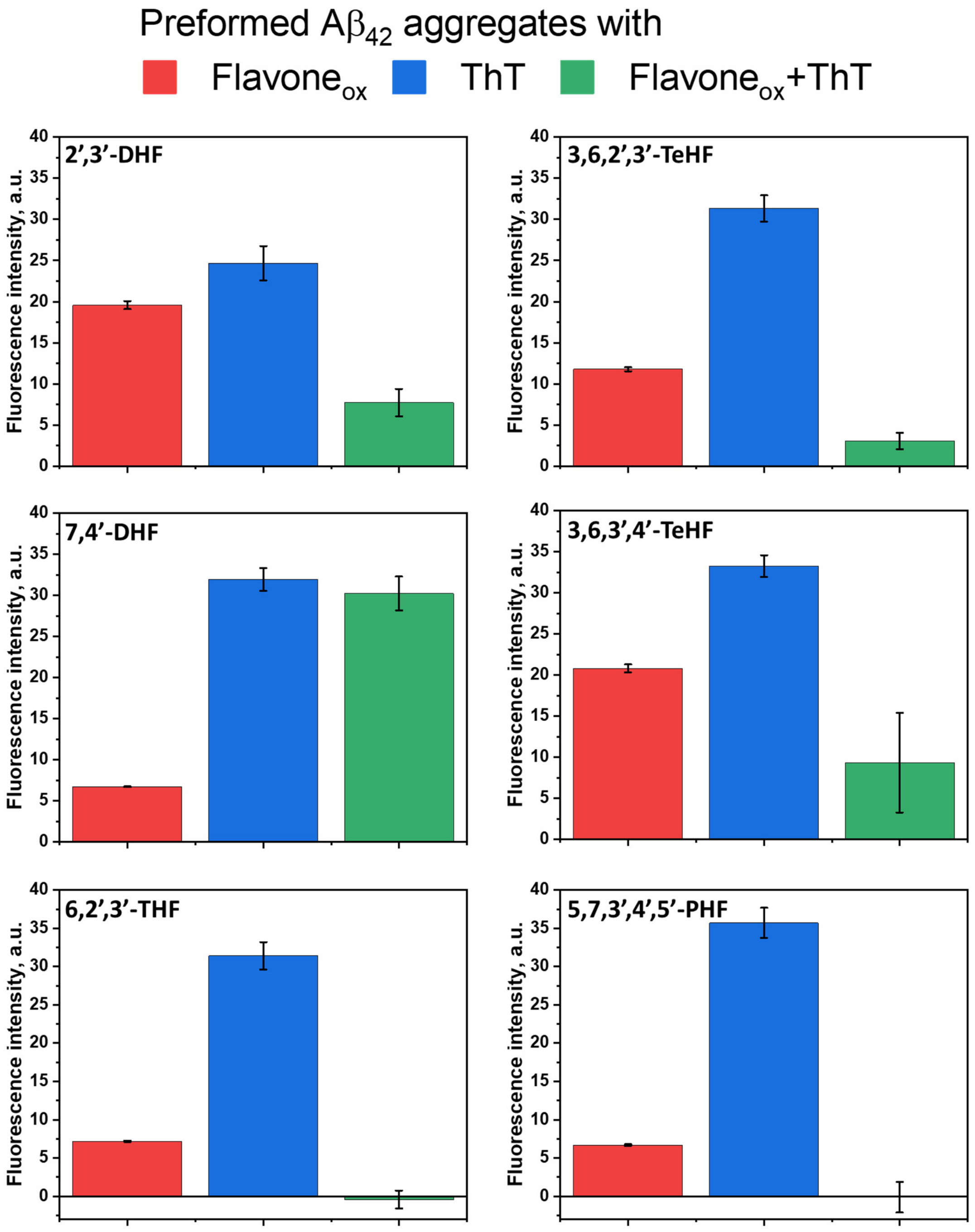

Figure S3. Fluorescence intensity values of preformed Aβ_{42} aggregates mixed with incubated flavone (red), ThT (blue) and incubated flavone with ThT (green). Intrinsic fluorescence of non-bound ThT and flavones was subtracted from their respective fibril-compound mixtures.

Table S1. Relative halftime and ThT fluorescence intensity values of Insulin and Amyloid-β aggregation.
* compounds spectra and aggregation halftime values are not shown in Figures 1-4.

		Non-oxidized flavone		Oxidized flavone			
**No.**	**Name**	**Insulin aggregation relative halftime**	**Insulin aggregation relative ThT fluorescence intensity**	**Insulin aggregation relative halftime**	**Insulin aggregation relative ThT fluorescence intensity**	**Amyloid-β aggregation relative halftime**	**Amyloid-β aggregation relative ThT fluorescence intensity**
**1**	3-hydroxyflavone	0.94 ± 0.08	1.08 ± 0.43	1.00 ± 0.15	0.74 ± 0.19	0.99 ± 0.09	1.01 ± 0.14
**2**	5-hydroxyflavone	0.88 ± 0.08	1.03 ± 0.36	1.07 ± 0.12	0.81 ± 0.23	0.95 ± 0.08	0.90 ± 0.12
**3**	6-hydroxyflavone	0.86 ± 0.07	1.00 ± 0.35	1.01 ± 0.20	0.78 ± 0.26	1.03 ± 0.09	0.96 ± 0.13
**4**	7-hydroxyflavone	0.91 ± 0.11	1.01 ± 0.45	0.93 ± 0.09	0.77 ± 0.29	0.93 ± 0.09	0.94 ± 0.14
**5**	2'-hydroxyflavone	0.93 ± 0.09	0.82 ± 0.28	1.08 ± 0.15	0.85 ± 0.29	0.56 ± 0.06	1.62 ± 0.23
**6**	3'-hydroxyflavone	0.90 ± 0.13	1.03 ± 0.64	0.99 ± 0.08	0.74 ± 0.31	1.04 ± 0.1	0.98 ± 0.16
**7**	4'-hydroxyflavone	0.84 ± 0.06	0.72 ± 0.22	0.95 ± 0.08	0.62 ± 0.29	1.00 ± 0.26	1.47 ± 0.96
**8**	3',5'-dihydroxyflavone	0.86 ± 0.06	0.83 ± 0.23	0.88 ± 0.06	0.71 ± 0.27	1.00 ± 0.09	1.00 ± 0.15
**9**	3,4'-dihydroxyflavone	0.87 ± 0.07	0.77 ± 0.22	0.95 ± 0.08	0.74 ± 0.23	0.98 ± 0.11	0.88 ± 0.13
**10**	5,6-dihydroxyflavone	0.88 ± 0.06	0.72 ± 0.28	1.96 ± 0.13	1.18 ± 0.58	1.07 ± 0.12	0.27 ± 0.04
**11**	7,8-dihydroxyflavone	0.92 ± 0.08	0.80 ± 0.29	3.73 ± 0.49	2.18 ± 1.29	1.05 ± 0.11	0.40 ± 0.08
**12**	6,7-dihydroxyflavone	0.83 ± 0.06	0.83 ± 0.30	0.99 ± 0.07	0.77 ± 0.25	1.02 ± 0.11	1.04 ± 0.46
**13**	6,4'-dihydroxyflavone	0.85 ± 0.06	0.80 ± 0.26	1.00 ± 0.11	0.82 ± 0.44	1.01 ± 0.10	0.96 ± 0.13
**14**	5,4'-dihydroxyflavone	0.90 ± 0.10	0.82 ± 0.32	0.94 ± 0.10	0.71 ± 0.27	0.79 ± 0.18	1.98 ± 0.41
**15**	3,2'-dihydroxyflavone	0.82 ± 0.05	0.82 ± 0.28	0.99 ± 0.07	0.68 ± 0.18	0.95 ± 0.10	0.80 ± 0.11
**16**	5,2'-dihydroxyflavone	0.85 ± 0.06	0.73 ± 0.24	0.93 ± 0.06	0.82 ± 0.22	0.70 ± 0.07	1.43 ± 0.24
**17**	6,2'-dihydroxyflavone*	0.91 ± 0.08	0.78 ± 0.24	0.86 ± 0.06	0.74 ± 0.23	0.82 ± 0.07	1.22 ± 0.17
**18**	7,2'-dihydroxyflavone	0.87 ± 0.06	0.67 ± 0.24	0.98 ± 0.09	0.68 ± 0.23	0.94 ± 0.16	1.20 ± 0.20
**19**	3,6-dihydroxyflavone	0.84 ± 0.07	0.92 ± 0.28	1.05 ± 0.14	0.89 ± 0.35	1.01 ± 0.11	1.03 ± 0.15
**20**	3,7-dihydroxyflavone	0.87 ± 0.07	0.99 ± 0.36	0.87 ± 0.08	0.75 ± 0.20	0.97 ± 0.09	1.10 ± 0.16
**21**	3',4'-dihydroxyflavone	0.85 ± 0.12	0.97 ± 0.29	2.06 ± 0.31	1.18 ± 0.25	1.01 ± 0.08	0.29 ± 0.04

		Non-oxidized flavone		Oxidized flavone			
**No.**	**Name**	**Insulin aggregation relative halftime**	**Insulin aggregation relative ThT fluorescence intensity**	**Insulin aggregation relative halftime**	**Insulin aggregation relative ThT fluorescence intensity**	**Amyloid-β aggregation relative halftime**	**Amyloid-β aggregation relative ThT fluorescence intensity**
**22**	2',3'-dihydroxyflavone	0.86 ± 0.06	1.02 ± 0.44	3.64 ± 0.50	1.36 ± 0.53	1.99 ± 0.22	0.05 ± 0.01
**24**	3,5-dihydroxyflavone	1.06 ± 0.11	1.13 ± 0.47	0.93 ± 0.09	0.78 ± 0.23	1.02 ± 0.11	0.91 ± 0.18
**25**	5,3'-dihydroxyflavone*	0.96 ± 0.11	1.03 ± 0.36	0.94 ± 0.08	0.78 ± 0.25	0.99 ± 0.06	0.91 ± 0.14
**26**	6,3'-dihydroxyflavone	0.98 ± 0.08	1.52 ± 0.65	1.03 ± 0.09	0.76 ± 0.21	0.94 ± 0.05	1.05 ± 0.16
**27**	7,3'-dihydroxyflavone	0.96 ± 0.09	1.48 ± 0.65	0.95 ± 0.12	1.18 ± 0.60	1.04 ± 0.06	1.00 ± 0.14
**28**	7,4'-dihydroxyflavone	1.03 ± 0.09	1.16 ± 0.45	0.96 ± 0.14	1.18 ± 0.65	1.04 ± 0.06	0.94 ± 0.12
**29**	3,3'-dihydroxyflavone	1.02 ± 0.09	1.24 ± 0.50	0.93 ± 0.11	1.05 ± 0.36	0.97 ± 0.06	0.99 ± 0.12
**30**	3,5,7-trihydroxyflavone (galangin)	0.95 ± 0.08	0.91 ± 0.33	0.96 ± 0.13	0.85 ± 0.33	1.01 ± 0.09	0.91 ± 0.18
**31**	6,2',3'-trihydroxyflavone	0.99 ± 0.07	1.07 ± 0.37	6.89 ± 0.78	8.33 ± 4.70	1.78 ± 0.19	0.04 ± 0.01
**32**	3,7,3'-trihydroxyflavone	0.96 ± 0.07	1.06 ± 0.45	1.04 ± 0.12	0.84 ± 0.41	1.04 ± 0.07	0.85 ± 0.11
**33**	3,6,3'-trihydroxyflavone	0.94 ± 0.07	1.04 ± 0.39	1.07 ± 0.12	1.22 ± 1.11	0.97 ± 0.08	0.86 ± 0.12
**34**	6,7,3'-trihydroxyflavone	0.98 ± 0.10	1.23 ± 0.43	0.87 ± 0.09	1.08 ± 0.36	1.01 ± 0.09	0.73 ± 0.12
**35**	3,6,2'-trihydroxyflavone	0.97 ± 0.08	1.34 ± 0.65	1.05 ± 0.12	0.87 ± 0.43	1.01 ± 0.07	0.74 ± 0.17
**36**	5,6,7-trihydroxyflavone (baicalein)	0.99 ± 0.08	1.02 ± 0.44	0.84 ± 0.08	0.88 ± 0.28	0.96 ± 0.10	0.99 ± 0.15
**37**	3,7,4'-trihydroxyflavone	0.93 ± 0.08	1.21 ± 0.51	1.18 ± 0.14	1.05 ± 0.42	1.04 ± 0.07	0.86 ± 0.14
**38**	7,8,2'-trihydroxyflavone	0.96 ± 0.07	0.76 ± 0.27	2.94 ± 0.32	2.45 ± 1.21	1.35 ± 0.15	0.13 ± 0.02
**39**	3,6,4'-trihydroxyflavone*	0.85 ± 0.07	1.01 ± 0.55	1.19 ± 0.21	1.34 ± 0.54	1.00 ± 0.07	0.76 ± 0.15
**40**	5,3',4'-trihydroxyflavone*	0.98 ± 0.08	0.96 ± 0.36	1.07 ± 0.18	0.96 ± 0.48	1.03 ± 0.09	0.83 ± 0.14
**41**	5,7,2'-trihydroxyflavone	0.90 ± 0.08	1.10 ± 0.63	0.91 ± 0.11	0.87 ± 0.41	1.10 ± 0.08	0.56 ± 0.07
**42**	6,3',4'-trihydroxyflavone	0.93 ± 0.11	1.15 ± 0.40	2.05 ± 0.47	1.26 ± 0.46	0.90 ± 0.08	0.25 ± 0.04
**43**	7,8,3'-trihydroxyflavone	0.91 ± 0.10	1.36 ± 0.47	3.18 ± 0.42	2.77 ± 1.97	1.35 ± 0.15	0.19 ± 0.03
**44**	7,8,4'-trihydroxyflavone	0.94 ± 0.09	1.32 ± 0.50	2.26 ± 0.47	1.59 ± 0.63	1.09 ± 0.10	0.39 ± 0.17
**45**	7,3',4'-trihydroxyflavone	0.92 ± 0.08	1.62 ± 0.71	1.31 ± 0.16	1.01 ± 0.29	0.98 ± 0.07	0.44 ± 0.05
**46**	3,3',4'-trihydroxyflavone	0.96 ± 0.09	1.10 ± 0.50	0.95 ± 0.09	1.16 ± 0.50	1.11 ± 0.09	0.25 ± 0.02

		Non-oxidized flavone		Oxidized flavone			
**No.**	**Name**	**Insulin aggregation relative halftime**	**Insulin aggregation relative ThT fluorescence intensity**	**Insulin aggregation relative halftime**	**Insulin aggregation relative ThT fluorescence intensity**	**Amyloid-β aggregation relative halftime**	**Amyloid-β aggregation relative ThT fluorescence intensity**
**47**	5,7,4'-trihydroxyflavone (apigenin)	0.96 ± 0.11	1.11 ± 0.43	1.08 ± 0.12	1.03 ± 0.26	0.92 ± 0.06	0.98 ± 0.09
**49**	3,6,2',4'-tetrahydroxyflavone	0.86 ± 0.11	1.43 ± 0.48	0.94 ± 0.09	0.89 ± 0.32	0.97 ± 0.07	0.51 ± 0.06
**50**	3,6,2',3'-tetrahydroxyflavone	0.89 ± 0.07	1.08 ± 0.44	1.01 ± 0.11	0.84 ± 0.20	1.08 ± 0.10	0.10 ± 0.01
**51**	7,3',4',5'-tetrahydroxyflavone	1.05 ± 0.09	1.28 ± 0.77	2.26 ± 0.26	1.77 ± 0.71	1.10 ± 0.09	0.25 ± 0.03
**52**	3,6,3',4'-tetrahydroxyflavone	0.94 ± 0.06	0.94 ± 0.36	4.65 ± 0.51	4.20 ± 1.58	1.50 ± 0.14	0.06 ± 0.01
**53**	3,7,8,2'-tetrahydroxyflavone	0.86 ± 0.06	0.97 ± 0.37	2.21 ± 0.60	1.98 ± 0.93	0.93 ± 0.08	0.48 ± 0.04
**54**	6,7,3',4'-tetrahydroxyflavone	0.90 ± 0.08	0.90 ± 0.31	0.87 ± 0.11	1.28 ± 0.43	1.09 ± 0.11	0.25 ± 0.10
**55**	3,5,7,4'-tetrahydroxyflavone (kaempferol)	0.87 ± 0.08	0.85 ± 0.35	0.80 ± 0.16	0.90 ± 0.32	1.03 ± 0.09	0.59 ± 0.05
**56**	5,7,3',4'-tetrahydroxyflavone (luteolin)	1.21 ± 0.13	1.25 ± 0.67	2.37 ± 0.32	0.98 ± 0.23	1.38 ± 0.16	0.17 ± 0.02
**57**	7,8,3',4'-tetrahydroxyflavone	1.01 ± 0.14	1.08 ± 0.41	3.60 ± 0.56	3.30 ± 0.71	1.46 ± 0.16	0.16 ± 0.02
**58**	3,5,7,2',4'-pentahydroxyflavone (morin)	1.01 ± 0.16	0.88 ± 0.34	1.02 ± 0.12	1.04 ± 0.40	0.92 ± 0.07	0.59 ± 0.05
**59**	5,7,3',4',5'-pentahydroxyflavone	0.84 ± 0.06	0.96 ± 0.50	7.06 ± 0.95	14.02 ± 7.92	1.35 ± 0.10	0.06 ± 0.02
**60**	3,6,2',4',5'-pentahydroxyflavone	1.00 ± 0.08	0.77 ± 0.32	4.43 ± 0.54	4.81 ± 3.95	1.24 ± 0.15	0.21 ± 0.05
**61**	3,5,7,3',4'-pentahydroxyflavone (quercetin)	0.87 ± 0.07	1.12 ± 0.39	3.26 ± 0.40	2.49 ± 0.97	1.00 ± 0.09	0.35 ± 0.05
**62**	3,7,3',4',5'-pentahydroxyflavone	0.82 ± 0.07	0.93 ± 0.34	2.71 ± 0.44	2.42 ± 1.20	1.04 ± 0.12	0.44 ± 0.07
**63**	3,5,7,3',4',5'-heksahydroxyflavone (myricetin)	0.82 ± 0.08	0.92 ± 0.46	8.19 ± 0.88	12.65 ± 2.17	1.34 ± 0.26	0.19 ± 0.02
**64**	3,5,7,8,3',4'-heksahydroxyflavone (gossypetin)	0.93 ± 0.09	0.99 ± 0.34	2.07 ± 0.33	1.36 ± 0.84	0.95 ± 0.09	0.65 ± 0.09
